# How long does telomerase extend telomeres? Regulation of telomerase release and telomere length homeostasis

**DOI:** 10.1007/s00294-018-0836-6

**Published:** 2018-04-16

**Authors:** Kazunori Tomita

**Affiliations:** Chromosome Maintenance Group, UCL Cancer Institute, University College London, London, UK

**Keywords:** Telomerase, Telomere length homeostasis, Shelterin, Chromatin, Replication, Processivity

## Abstract

Telomerase, the enzyme that replenishes telomeres, is essential for most eukaryotes to maintain their generations. Telomere length homeostasis is achieved via a balance between telomere lengthening by telomerase, and erosion over successive cell divisions. Impaired telomerase regulation leads to shortened telomeres and can cause defects in tissue maintenance. Telomeric DNA is composed of a repetitive sequence, which recruits the protective protein complex, shelterin. Shelterin, together with chromatin remodelling proteins, shapes the heterochromatic structure at the telomere and protects chromosome ends. Shelterin also provides a foothold for telomerase to be recruited and facilitates telomere extension. Such mechanisms of telomere recruitment and activation are conserved from unicellular eukaryotes to humans, with the rate of telomere extension playing an important role in determining the length maintained. Telomerase can be processive, adding multiple telomeric repeats before dissociating. However, a question remains: how does telomerase determine the number of repeats to add? In this review, I will discuss about how telomerase can monitor telomere extension using fission yeast as a model. I propose a model whereby the accumulation of the Pot1 complex on the synthesised telomere single-strand counteracts retention of telomerase via chromatin proteins and the similar system may be conserved in mammals.

## Main

Telomerase plays a critical role in telomere maintenance, but its regulation at the telomere remains to be established. Telomeres are non-coding guanine (G)-rich DNA repeats located at the termini of linear chromosomes. Telomeric DNA terminates in a G-rich single-stranded 3′ end or G-overhang. They function to stabilise and protect chromosome ends. This is achieved by the recruitment of the telomeric protein complex, shelterin (de Lange 2005). Conversely, telomere attrition occurs after the DNA replication, meaning that telomeres progressively shorten through each division of the cell. Before becoming critically short and compromising shelterin functions, cells permanently arrest cycling in a phenomenon called ‘replicative senescence’. Some stem cells and progenitor cells express the specialised reverse transcriptase, telomerase, to add telomeric repeats and delay replicative senescence. When telomere addition by telomerase, and erosion of telomeric DNA are balanced, cells achieve a homeostatic telomere length (Hug and Lingner 2006). This telomere length homeostasis is observed in unicellular eukaryotes and germ cells, permitting telomeres to be maintained to future generations. The conserved function of telomerase and the shelterin complex make organisms such as fission yeasts useful models to study fundamental aspects of telomere maintenance (Armstrong and Tomita 2017).

Telomerase activity is tightly controlled at multiple levels, from the expression of telomerase components to the appropriate functioning of assembly factors required to recruit and activate telomerase at the telomeres (Armstrong and Tomita 2017; Churikov et al. 2013; Schmidt and Cech 2015). Defects in any of these process lead to loss of telomeric repeats or the maintenance of short telomeres. In unicellular organisms, loss of telomerase activity results in ever shorter telomeres (EST) phenotype and the catastrophic cell death. However, destabilised telomeres caused by telomere erosion can rarely activate alternative lengthening of telomere (ALT) pathways, wherein homology-directed recombination/replication copies telomeric repeats via strand invasion to inter/intra-chromosome ends (Gadaleta et al. 2016; Lue and Yu 2017). Alternatively, impaired telomerase activity can be overcome by change in transcription levels, caused by selective aneuploidy (Millet and Makovets 2016). Therefore, telomerase must be correctly controlled to maintain genome stability. In humans, mutations that impair telomerase activity increase the risk of many diseases and, in severe cases, cause development/regeneration-related disorders (Blackburn et al. 2015). These defects are presumably originated from replicative senescence of stem cells and impaired tissue regeneration.

Telomerase is recruited to the telomeres via shelterin or other telomere located proteins (Cdc13 in budding yeasts, TPP1-TIN2 in mammals and Tpz1-Ccq1 in fission yeasts) (Armstrong and Tomita 2017). The recruited telomerase relocates to the end of a single-stranded G-overhang by hybridising with its RNA template, allowing the reverse transcriptase component to add new repeats. The interaction between telomerase and shelterin and/or the formation of the telomeric DNA/telomerase RNA hybrid retain telomerase at the telomere, permitting the processive extension of telomeres (Greider 1991; Wang et al. 2007; Xin et al. 2007). Telomerase preferentially targets the shortest telomeres for extension, which ensures that an average telomere length is maintained across all chromosomes (Hug and Lingner 2006).

Telomere elongation is observed only during S-phase. At the end of S-phase, telomerase access is blocked by the DNA-replication complex CST. This comprises of CTC1, STN1 and TEN1 in mammals and Stn1 and Ten1 in budding and fission yeasts (Chen and Lingner 2013). In budding yeast, Stn1-Ten1 binds to Cdc13 to block the interaction of telomerase (Churikov et al. 2013). A similar mechanism has been proposed in mammals and fission yeast, where in the CST complex associates with TPP1 and Tpz1, respectively, transiently in late S-phase. Furthermore, the CST complex recruits the primase-polymerase α complex to fill the complementarily strand of the newly synthesised telomere by telomerase (Feng et al. 2017; Grossi et al. 2004). Outside S-phase, telomeres form a closed structure, blocking telomerase access known as a ‘non-extendible state’ (Hug and Lingner 2006). This is possibly via the accumulation of suppressive proteins that bind the double-stranded telomere DNA (the classical counting mechanism) (Marcand et al. 1997), the folding of the 3′ G-overhang by the shelterin bridge (between single-strand and double-strand telomeres) (Jun et al. 2013), or via a t-loop formation, in which 3′ G-overhang end displaces and anneals to its double-stranded telomere (de Lange 2005). All these closed formation models involve the shelterin proteins.

How does telomerase dissociate from the telomere? When telomerase is released, a newly elongated telomere can be subjected to further rounds of telomere extension during S-phase, implying that the telomere dissociation is reversible. Hence, a distinct mechanism from the telomerase termination processes, such as reforming of t-loops or recruitment of the CST complex, are expected to occur. In budding yeast, the DNA helicase Pif1 preferentially releases telomerase RNA by unwinding the DNA/RNA duplex (Bochman et al. 2010; Sabouri 2017; Stinus et al. 2018). Pif1 is well-conserved in mammals and expected to perform a similar job. However, how and when these DNA helicases are recruited to the end of telomere are not known. Another mechanism that dissociates telomerase from the telomeric proteins is also expected.

How is telomere extension monitored? Processive telomerase is thought to be released once the telomere is sufficiently elongated. This means that a counting mechanism for telomere extension is likely to exist. The synthesised telomeric DNA can form a G-quadruplex structure. However, such secondary structure would be unwound by the binding of the RPA (replication protein A) complex, which facilitates telomerase activity (Audry et al. 2015). RPA is replaced by the single-strand DNA binding shelterin protein, Pot1 (Flynn et al. 2011; Ray et al. 2014). Hence, I predict that accumulation of the Pot1 complex on the synthesised telomere G-strand DNA serves to release telomerase, reminiscent of the classic counting mechanism (Marcand et al. 1997). Pot1 interacts with TPP1 in mammals and Tpz1 in fission yeast. Therefore, the Pot1 complex at the 3′ end could attract telomerase for further rounds of extension.

**Fig. 1:**
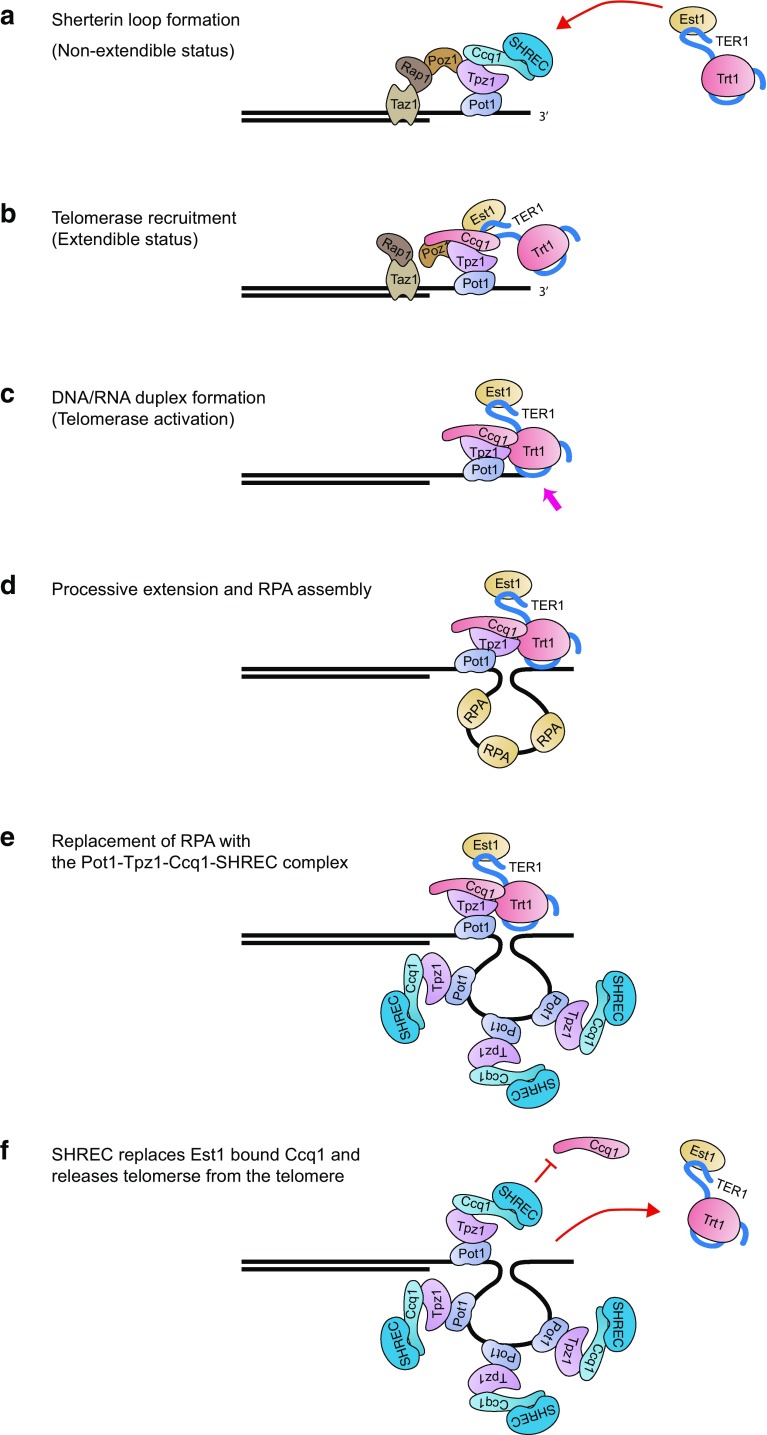
Proposed model for telomere extension and telomerase turn over in fission yeast. **a** Non-extendible conformation of shelterin stabilises 3′ end of the telomere. Shelterin is comprised for Pot1, Tpz1, Ccq1, Poz1, Rap1 and Taz1 that binds to the double-stranded telomeric DNA. Ccq1 may interact with SHREC. Est1 can be recruited to Ccq1 when it becomes phosphorylated. **b** The interaction between Ccq1 and Est1 results in release of SHREC from Ccq1 and the telomere becomes an ‘extendible state’ (dissolution of the shelterin bridge; Poz1 is dissociated from Rap1 as an example in this diagram). **c** Trt1 associates with Tpz1 and Ccq1 to promote telomerase activation. The RNA template of TER1 hybridises with 3′ end of the telomere overhang (Arrow: DNA/RNA hybrid formation). Taz1, Rap1 and Poz1 are omitted from the diagram. **d** Stabilised telomerase adds telomeric repeats to the G-rich strand. RPA binds to the synthesised DNA. **e** The Pot1-Tpz1-Ccq1 complex replaces RPA. Ccq1 may recruit SHREC. **f** Accumulation of the Pot1-Tpz1-Ccq1-SHREC complex on the telomere overhang releases the telomerase-associated Ccq1 complex from the telomere. The Pot1 complex at the 3′ end of the G-overhang can recruit telomerase (back to **a**)

The clue as to how telomere extension is monitored might be found in fission yeast. Fission yeast Pot1 stably forms a complex with Tpz1 and Ccq1. Together with Tpz1, Ccq1 has multiple roles in telomerase regulation, from its recruitment, activation and release (Armstrong et al. 2014, 2018; Tomita and Cooper 2008). Telomerase recruitment is stimulated by the phosphorylation of Ccq1, which increases the affinity between Ccq1 and the telomerase subunit, Est1 (Moser et al. 2011; Webb and Zakian 2012). Our recent study suggested that Ccq1 also counteracts telomerase retention at the telomere via the chromatin remodelling NuRD complex, SHREC (Armstrong et al. 2018). Interestingly, SHREC appeared to bind Ccq1 that is not associated with Est1, thereby separating active (Ccq1-Est1) and inactive (Ccq1-SHREC) forms. SHREC controls telomere length only when telomerase is retained at the telomeres, demonstrated by the artificial recruitment of telomerase. Hence, SHREC appeared to have a role in release of telomerase from the telomere rather than in the telomerase recruitment process. This activity requires complex formation with Ccq1 and Tpz1. Therefore, I proposed a model, whereby Ccq1-SHREC is recruited to a newly synthesised telomere G-strand via Pot1 and releases Ccq1-Est1, along with telomerase, from the telomere (Fig. 1). Simultaneously, DNA/RNA helicases would remove telomerase from the DNA end. As SHREC does not suppress the telomerase recruitment process, Ccq1 at the tip of the G-overhang can be phosphorylated and telomerase could be re-directed again, until the telomere becomes long enough to form a ‘non-extendible state’.

In humans, the Ccq1 equivalent, TIN2, associates with telomerase as well as a heterochromain protein 1 (HP1), and these association sites appeared to be overlapped (Canudas et al. 2011; Yang et al. 2011). Interestingly, overexpression of HP1 proteins leads to shortening of telomeres (Sharma et al. 2003). These data imply a mutually exclusive interaction of telomerase and HP1 with human TIN2, supporting the fission yeast telomerase release model, whereby TIN2-HP1 replaces TIN2-telomerase at the telomere. In budding yeast, the inactive form of telomerase associates with the chromatin protein, Sir4, at the telomere. When telomerase is activated, the Cdc13-Est1 complex replaces the Sir4 complex for telomerase association (Chen et al. 2018). Thus, a role of chromatin proteins in mediating telomerase inactivation might be conserved among species.

In this review, I summarised the potential function of the chromatin proteins in telomerase control. Mutually exclusive interaction of the chromatin proteins to the telomeric proteins and/or telomerase can counteract telomerase retention at the telomere. Like the processes for telomerase recruitment and blocking, release of active telomerase contributes to telomere length homeostasis. Further investigation of two key areas is required to establish a high-resolution picture of telomerase regulation: how the newly synthesised G-strand is organised during telomerase catalytic process and how telomerase activity is terminated following processive telomere extension.
